# GENDER DIFFERENCES IN ASSOCIATIONS OF SLEEP INTRAINDIVIDUAL VARIABILITY AND MILD COGNITIVE IMPAIRMENT

**DOI:** 10.1093/geroni/igad104.1286

**Published:** 2023-12-21

**Authors:** Yuqi Shen, Linying Ji, Jennifer Graham-Engeland, Carol Derby, Orfeu Buxton

**Affiliations:** Pennsylvania State University, State College, Pennsylvania, United States; Montana State University, Bozeman, Montana, United States; Pennsylvania State University, State College, Pennsylvania, United States; Albert Einstein College of Medicine, Bronx, New York, United States; Pennsylvania State University, State College, Pennsylvania, United States

## Abstract

Previous studies suggest that cognitive function in older adults is related to sleep, with females more vulnerable to poor sleep. Little is known about the relationship between cognition and intraindividual variability of sleep (IIV). We examined the cross-sectional relationship between sleep IIV and mild cognitive impairment (MCI) by gender using data from the Einstein Aging Study. We hypothesize that individuals with MCI have a greater IIV in daily sleep characteristics, and these associations will be stronger for females. Eighty-nine men (age = 76.81±4.73, 61% White, 28% Black, 27% MCI) and 197 women (age = 76.60 ± 4.93, 40% White, 46% Black, 29% MCI) were included in the analysis. Individuals completed 2 weeks of actigraphy (mean ± SD = 15.7 ± 1.1 days), and the Montreal Cognitive Assessment, and MCI was based on the Jack/Bondi criteria. Sleep IIV was estimated as within person standard deviation of nighttime sleep duration, 24-hour sleep duration, wake after sleep onset, and nighttime sleep midpoint. Linear regression models evaluated associations between sleep IIV and MCI status separately by gender, controlling for BMI, age, ethnicity/race, nocturnal hypoxemia, Oxygen Desaturation Index ≥15. Among women, having MCI was associated with greater IIV in nighttime sleep midpoint (b=0.19, p=.002), and in 24-hour total sleep duration (b=13.10, p = .047) with a similar trend in nighttime sleep duration (p = .050). No significant associations were found among men. Findings suggest the importance to keep considering gender in work to understand how sleep variability relates to MCI status in older adults.

